# How do European citizens prioritize environment and climate issues? The role of the value “Respect for the planet” in combination with education and cultural background

**DOI:** 10.1007/s13280-026-02350-w

**Published:** 2026-03-01

**Authors:** Joop de Boer, Harry Aiking

**Affiliations:** Institute for Environmental Studies, Vrije Universiteit Amsterdam, De Boelelaan 1111, 1081 HV Amsterdam, The Netherlands

**Keywords:** Cultural autonomy, Ideological values, Issue salience, Planet Earth, Tertiary education

## Abstract

This study investigates whether citizens’ support of environment and climate policies can be stimulated by targeting their relationship to Planet Earth, using “Respect for the planet” as a political value. The study is based on seven Eurobarometer surveys across the period 2019 to 2024, supplemented by country-level data. Endorsing the value Respect for the planet was associated with a higher priority given to environment and climate issues, and this association was stronger in combination with an advanced education and a country’s cultural context that values individuals’ autonomy (versus their embeddedness in social networks). These results were relatively independent of the variable left–right political placement, which was added for comparison. Hence, Respect for the planet has the potential to become a core political value that, in combination with advanced education and appropriate cultural conditions, can support the pursuit of environment and climate objectives, such as the legitimation of policy changes.

## Introduction

In view of the serious transgressions of planetary boundaries, many scientists agree that environment and climate issues have to get far more priority over other issues in the policies and actions of governments and citizens (Rockström et al. 2024). To stimulate the support of citizens, several scholars emphasize that scientific facts are too impersonal and that communications with citizens should appeal more to the environmental imagination of individuals and, in particular, to their feelings, beliefs, and understandings regarding “Planet Earth” (Feitelson 1991; Jasanoff 2010; Devine-Wright et al. 2015). The present study investigates whether citizens’ support of environment and climate policies can be stimulated by targeting their relationship to Planet Earth. Specifically, we examine the potential of the value Respect for the planet to become a core political value and demonstrate the roles thereby of education, left–right political placement, and cultural context.

The concept of value is used in social, political, and legal disciplines with slightly different meanings (e.g., Frischhut 2022). A social-psychological approach conceptualizes broad, *personal values* as desirable goals, of different importance, that serve as guiding principles and motivational forces in people’s life (Sagiv and Schwartz 2022). Examples include the importance of achieving personal success or the importance of protecting the welfare of *all* people and for nature. In the area of political life, there are *political values* of which the *core political values* provide overarching normative principles and belief assumptions about government, citizenship, and the nation’s society that shape policies and action (McCann 1997; Schwartz et al. 2010), such as by referring to the importance of Human rights. According to Schwartz et al. (2010), the personal values may influence political choice through their influence on core political values. The personal and political values can be distinguished from *cultural value orientations,* i.e., conceptions of what is good and desirable among people in a society, which are manifested in the behavior of social institutions as well as individuals (Schwartz 2014, 2015), such as the importance of individual autonomy in a country. The cultural value orientations form the context of people’s behavior.

In the development of the European Union (EU), political values, such as Democracy and The rule of law, are political ideals that form the foundation of the European treaties (Foret and Calligaro 2018; Frischhut 2022; Polak 2023). The values are used in all kinds of political interactions, including interactions with citizens. A specific tool for the interactions with citizens is the organization of European surveys (the “Eurobarometer”), which gather the opinions of citizens in all Member States and often in some other countries also. The content of the surveys is policy-relevant, although the specific link between survey questions and particular policy areas is not explicitly explained in the reports (Haverland et al. 2018). Several Eurobarometers include a question that asks the participating citizens to make a selection out of a list of political values; since 2019 this list has been extended with the value Respect for the planet (Frischhut 2022, p. 220). The relevance of this extension, which was meant to add to the human-centric values a more bio-centric approach (Frischhut 2022, p. 248), has not been analyzed in a systematic and theory-based way up till now.

The present study aims to fill this gap, taking into account that values are, in fact, abstract and general descriptions that need to be interpreted in daily practice (Polak 2023, p. 47). It is critical, therefore, to consider how the value relates to a specific political issue, in combination with conditions that may provide people with knowledge and skills that guide their interpretations and actions. An adequate dependent variable is citizens’ choice of the most salient issue for action and policy-making (Dennison 2019; Crawley et al. 2022). More specifically, then, our aim is to assess whether and under what conditions citizens who select Respect for the planet as one of their important political values are more likely than others to select climate and environment issues (henceforth “*green issue salience*”) as priority issues for policy action. Although the relationship between selected value and selected issue for action is not very interesting in itself, it is important to assess whether and how this relationship varies with conditions that stimulate and empower citizens in developing thoughts and actions, such as having tertiary education (Noy and O’Brien 2019; Welsch 2022; de Boer and Aiking 2024) and the economic-cultural context in which they live (Inglehart 2006; Schwartz 2014). As issue salience also varies with citizens’ *left–right political placement* (Crawley et al. 2022), which has proven capable of incorporating many types of political conflict (Knutsen 1995; Weber and Saris 2015), this variable is used for comparative purposes.

We base our analysis on seven Eurobarometer surveys that included Respect for the planet as one of the values to choose from. It is the only value in the list that refers to the natural environment. The survey items that measured the salience of green issues among the participants belong to the standard Eurobarometer questions; these were used in earlier research to predict the green vote share at regional level (Hoffmann et al. 2022). The dataset involved 249,040 participants (15 years and older) in 38 European countries across the period 2019 to 2024, although with irregular intervals. The countries are members and non-members of the EU and belong to different economic-cultural zones with different political and religious historical backgrounds. The zones are Northwestern (Protestant) Europe, Central (Roman Catholic) Europe, Southeastern (Orthodox) Europe, and Former Communist Europe (Protestant, Catholic, or Orthodox) (Huntington 1993; Inglehart 2006; Schwartz 2014; Akaliyski et al. 2022).

In short, the paper aims to provide three contributions to the literature. First, it introduces Respect for the planet as a recently introduced political value and examines whether citizens’ choice of this value is associated with a higher priority of green issues. Second, it analyzes whether this association varies in combination with secondary and tertiary education, controlling for left–right political placement. Third, it shows how this association is influenced by the economic-cultural zones in Europe, including the gap between countries with and without a Communist inheritance.

## Theoretical framework

### Respect for the planet as a political value

This section briefly clarifies some theoretical aspects of Respect for the planet, using the literature about personal and political values. Much work in this field has been done to identify organizing principles that constrain and structure values. A wide variety of personal values can be condensed into a circular continuum of compatible (vs conflicting) motivations. One of the axes of the circle is the opposition between “self-enhancement values” (i.e., the importance of achieving personal success) and “self-transcendence values,” which include values that refer to the importance of the protection for the welfare of all people and for nature (Schwartz and Cieciuch 2022). As personal values may influence political choice through their influence on core political values (Schwartz et al. 2010), the self-transcendence value of nature protection may be associated with Respect for the planet.

Unlike the circular formation of the personal values, however, there is, as yet, no clear consensus regarding the number and content of core political values in modern democracies (Schwartz et al. 2010). To a certain extent, the endorsement of political values may correspond with different positions across the left–right political dimension of progressives versus conservatives. This involves differences between, on the one hand, political progressives who favor openness, are tolerant to ambiguity, and have a desire for change and, on the other hand, political conservatives whose values are concerned with preserving the social order and status quo (Waytz et al. 2019). However, liberalism–conservatism (or left–right) does not appear to be the only organizing principle that constrains and structures the core political values (Schwartz et al. 2010). In other words, political values are not specifically embedded in a structure of other values, and they may be related to but are distinct from the left–right political spectrum.

A special point to be noted is that Respect for the planet is the only political value that has a spatial dimension as it refers to a specific place. The explicit link with the planet may have emotional connotations, and emotions can increase the motivational force of a value (Sagiv and Schwartz 2022). There is a distinct field of research that studies the emotional bonds that an individual may establish with specific places, including the emotional and identity-related aspects of people–place relations (Altman and Low 1992). The concept of “global place attachment” has been suggested to support the argument that people can develop relations of belonging and stewardship to the whole Earth, and not just to the place (e.g., city or neighborhood) where they live (Feitelson 1991; Devine-Wright et al. 2015). A recent Australian survey measured individuals’ sense of belonging to “The Earth/The whole world” and indicated that global place attachment is associated with an understanding of climate issues that agrees with current scientific insights (Devine-Wright et al. 2015). In line with this study that showed a relationship between global place attachment and an informed climate opinion, we expect a relationship between Respect for the planet and green issue salience.

#### The importance of education

Education, in particular at tertiary level, is often considered to be of crucial importance for addressing environment and climate issues, such as the social acceptance of policy measures (Meyer 2015; Lee et al. 2024; Eckert et al. 2025), and, in general, the pursuit of the United Nations Sustainable Development Goals (SDGs) (Fehlner 2019; Žalėnienė and Pereira 2021). As evidence from Europe demonstrates (Meyer 2015), education may provide people with knowledge and skills as well as socialization experiences to be concerned about overall social welfare, including protection of the environment.

Recently, salient references to the future of the planet and the people were made by the climate protests of students in various countries, which started in 2018 and became a worldwide phenomenon by early 2019 (Boulianne and Ohme 2022; Svensson and Wahlström 2023). International research reports that the school strikers followed secondary or tertiary education (Svensson and Wahlström 2023), but other actions (e.g., by Extinction Rebellion) were more characterized by those with tertiary education who participated in university student life (Pickard 2022). The new climate movements show a strong emphasis on the clarity of scientific findings about climate change, and a strong emotional emphasis on urgency, calling on politicians to act, here and now (Buzogány and Scherhaufer 2023; Yearley 2024). Although young climate activists have drawn attention to the role of the youth, data from various countries indicate that anthropogenic climate change awareness and concern have increased over the 2009–2021 period among all age groups (Lorenzini et al. 2021; Milfont et al. 2021), although the youth may demonstrate stronger emotional engagement (Poortinga et al. 2023). Apart from that, the students’ climate protests showed a level of a green issue salience that may have been shared by other age groups with a similar education level.

However, research has shown that the positive impacts of education are interfered with by political processes in affluent countries and polarization along the left–right dimension (Smith and Mayer 2019; Czarnek et al. 2021). The role of education nowadays seems to be that it provides people with knowledge and skills to bring their beliefs and actions in line with their political positions (or those of their peers), which may have positive or negative consequences for their responses to climate and environment issues (Kahan et al. 2012; Czarnek et al. 2021; Hornsey 2021; Welsch 2022). We expect a similar moderating impact of education on the relationship between the political value Respect for the planet and green issue salience.

### Influences by the economic-cultural zones in Europe

This section follows Huntington (1993) who highlighted differences in the broad cultural heritage of a society, which mainly has a background in its religious history—in Europe, this now includes Communist heritages. Building on this work, value theorists (Schwartz 2014) and modernization theorists (Inglehart 2006) have shown the importance of values at the level of countries and economic-cultural zones. These theorists have drawn similar maps that are based on the positions of European (and other) countries in terms of their cultural values (Schwartz 2014) or types of values (Inglehart 2006).

Schwartz’s (2015) cultural value orientations address the place of individuals in the group—emphasizing “Autonomous” ways of living (versus “Culturally embedded” ways of living) and “Egalitarianism” (versus “Hierarchy”)—dimensions, which are linked to economic and social progress in a country. In the Protestant countries of Northwestern Europe, the importance of these cultural values has—as a result of many reciprocal causal processes—led to policies and practices that produce more National affluence and more Gender equality in social, economic, and political life (Schwartz 2015). The importance of the autonomous and morally equal ways of living appears to decrease from Northwest to Southeast Europe (Schwartz 2014). In terms of Inglehart and Baker (2000), the citizens of affluent societies place increasing emphasis on quality-of-life, environmental protection, and self-expression over survival values and traditional values, although this does not mean that they are equally aware of their environmental footprint abroad (Presberger et al. 2023) (to be reiterated in the Discussion).

The cross-national differences may also affect the degree to which pro-environmental or pro-climate variables are related to each other and to third variables. It should be acknowledged, however, that the cross-cultural aspects of environmental psychology are yet not fully developed (Tam and Milfont 2020). Some associations are stronger in affluent countries and in Nordic countries than in others (Hoffmann et al. 2022; Czarnek et al. 2025). These cross-level interactions may, in particular, involve variables that stimulate and empower citizens in developing thoughts and actions, such as education (Noy and O’Brien 2019; de Boer and Aiking 2024). In addition, there are situation-specific factors, such as the intensity of political conflicts in countries with higher stakes to gain or to lose as a result of climate policies. This may explain that the variable political left–right placement shows larger associations with climate-related opinions in higher affluent countries (Smith and Mayer 2019; Hoffmann et al. 2022; Czarnek et al. 2025) than in countries where climate-related issues are still neutral.

The modernization trajectories of many European countries were affected by the lasting effects of a Communist regime (Inglehart and Baker 2000). There are FC countries with a Catholic or Protestant tradition that joined the EU, and FC countries with a Christian-Orthodox tradition, most of which remain outside the EU (Akaliyski et al. 2022). Important for the present study is that the left–right dimension has a different meaning in the FC countries (McCright et al. 2016; Baranowski et al. 2025) and that the salience of green issues in these countries remains low (Smith and Mayer 2019; Fraembs and Drobnič 2024). This implies that we cannot use the variable left–right political position in FC countries, that we may expect differences in the reliability of the measure of green issue salience, and that these countries may only play a small role in this study.

## Materials and methods

### Fieldwork and data collection

The surveys were carried out by research firm Kantar, commissioned by the EU, in the 27 Member States of the EU, five candidate countries, plus Bosnia and Herzegovina, Kosovo, Moldova, Norway, Switzerland, and the United Kingdom. Descriptions of the fieldwork and the questionnaires are connected with the datasets of the Eurobarometers (European Commission 2020, 2021, 2022, 2023, 2024a; b, c). The surveys in 2021 and 2022 were conducted during the COVID-19 pandemic, which necessitated a number of adjustments as face-to-face interviews had to be complemented with online interviews. Overall, 90% of the participants were interviewed face-to-face and 10% online. Potential mode effects were made visible in the analysis.

#### Individual-level variables

The dependent variable *green issue salience* was derived from the question of what the participants believe are the two most important issues that affect (1) their country, (2) themselves personally, and (3) the EU member states collectively, respectively. The participants saw three times a list of about 15 issues, which were presented in a rotating order with small variations across the three questions. In each list, “the environment and climate change” was the only green issue. Other work has shown that it is typical for this issue that all three entities (country, themselves, and the EU) are consistently related (Bevan et al. 2016). Hence, a multi-item measure of salience could be planned as the dependent variable. The EU-version was also asked for in non-member states and, therefore, EU membership was taken as a country-level covariate. However, the EU-version was not asked for in all surveys throughout the period and the participants who did not get the question had to be left out of the analysis.

*Respect for the planet* was a value that had to be chosen from a list of 13 values plus the options “none” or “don't know.” The presented values reflected to a certain degree the ideological values on which the European Union (EU) is founded, such as Democracy and Respect for human rights (Donders 2020). The participants were asked to indicate the most important values for them personally, with maximally three answers. Respect for the planet was a dichotomous variable (chosen/not chosen); the other choices were not further analyzed to avoid unwanted dependencies between the values.

The measure of *left–right political placement* was based on a standard question. The participants were asked to place their views on a scale with ten categories (plus don’t know or refusal). In the analyses, a political placement dummy was created to separate the participants who placed themselves on the 10-pts scale from those who did not. For reasons of presentation, the ten categories were reduced to five.

*Age left education* referred to how old the participants were when they stopped full-time education. This measure was used as a proxy for educational attainment, assuming that participants who left their education at the age of 23 years or older had tertiary education (Simister 2014) and those who left at the age of 17 to 22 years had secondary education. The answers were categorized as “at the age of 16 or below,” “17–22,” and “23 or above.” Those who were still studying were classified based on their current age.

Individual-level control variables were *gender* (three gender categories, covered by a dummy for women and a dummy for gender diverse persons), *age* (six categories), *living in a rural or urban environment* (three categories), and *interview mode* (two categories).

#### Country-level variables

The positions of the countries in the *economic-cultural zones* (3 categories from Northwestern to Southeastern Europe) were chosen from the map presented by Schwartz (2014). The measure of *National affluence* was taken from the World Bank and refers to Gross National Income (GNI) per capita based on purchasing power parity (PPP) in constant 2017 international dollars, averaged over the years 2017–2020 (and log transformed). As National affluence is closely correlated with the social and economic aspects of gender equality (Schwartz 2015), only political equality was included. *Women’s representation* was the percentage of seats held by women in national parliaments and governments, taken from Eurostat and averaged over five years (2017–2021). Additional country-level variables were *year of the survey* (seven categories), FC heritage, and EU membership.

### Analyses

A preliminary analysis examined the correlations between the country-level variables to determine the variables for the multilevel analysis. Next, Cronbach’s alpha was calculated to check the consistency between the three items of issue salience in non-FC and FC countries, respectively. This consistency is crucial for the reliability of the summed item score. As the alpha-value tends to underestimate the internal consistency of scales shorter than 10 items, the mean inter-item correlation was used as an alternative (Taber 2018). This measure should fall in the range 0.15–0.50, dependent on the generality (closer to 0.15) or specificity (closer to 0.50) of the target construct (Clark and Watson 1995).

The multilevel analysis with green issue salience as the dependent variable was performed through linear mixed regression modeling, which considered individuals to be nested within countries and survey years. The analysis was performed in several models that added variables to the prediction. As some of the variables were measured in quite different units, they were all standardized, except the dichotomous variables and the variable survey years. It should be noted that the very large, pooled sample limits the usefulness of statistical significance (very small correlations are still significant). Therefore, we only report the coefficients of the control variables in the Table if their absolute value is ≥ 0.10 (see Steenkamp 2019), except if there are special reasons to make an exception. The significant interactions were visualized by bar charts. All analyses were performed with SPSS 29 for Windows.

#### Preliminary analyses of the country differences

The variable economic-cultural zone played a central role in the correlations between the country-level variables. Compared to the Northwest the countries in the Southeast were much lower in National affluence (*r* = − 0.78, *N* = 38, *p* < 0.001), and Women’s representation (*r* = − 0.40, *N* = 38, *p* < 0.05); they had more often a Communist inheritance (*r* = 0.47, *N* = 38, *p* < 0.01), and were less often an EU member (*r* = − 0.32, *N* = 38, *p* < 0.05). Taking these correlations into account, it was decided that economic-cultural zone and EU membership would be the country-level variables in the multilevel analysis.

## Results

This section first presents the findings about Respect for the planet and green issue salience, also considering whether the results in non-FC and FC countries can be examined in the same analysis. Subsequently, the focus is on the moderating impacts of education and zone.

### Respect for the planet and green issue salience

The value Respect for the planet was chosen by 16% in the non-FC countries and by 7% in the FC countries. The differences in green issue salience between the non-FC and the FC countries are shown in Table 1. In the non-FC countries, the individual items scored higher and the internal consistency between them was higher than in the FC countries. What did not differ was that the importance for the EU was mentioned more often than the importance for the country and for the participants personally. In the non-FC countries, the items formed an adequate scale (alpha = 0.60). However, in the FC countries the internal consistency of the three items was marginal (i.e., the mean inter-item correlation was 0.14 and just below the 0.15 mentioned in the literature). The difference in the reliability of the summed item scores is a reason to keep the analyses apart. Those in the non-FC countries who chose Respect for the planet scored 0.71 SD higher than the others on green issue salience; in the FC countries this difference was 0.41. Hence, the full analysis could be carried out in the non-FC countries; a more limited approach (without left–right political placement) was possible in the FC countries.

**Table 1:** Measure of green issue salience in non-FC countries and FC countries

	Non-FC countries	FC countries
*Scale items*		
Green issues chosen as important for their country	19%	6%
Green issues chosen as important for themselves personally	16%	6%
Green issues chosen as important for the EU member states collectively	24%	11%
*Scale statistics*		
Cronbach’s alpha	0.60	0.31
Mean inter-item correlation	0.33	0.14
Mean summated score	0.58	0.22
*SD*	0.88	0.51
*N* =	124 044	110 684

### Moderating impacts of education and economic-cultural zone (non-FC countries)

**Fig. 1:**
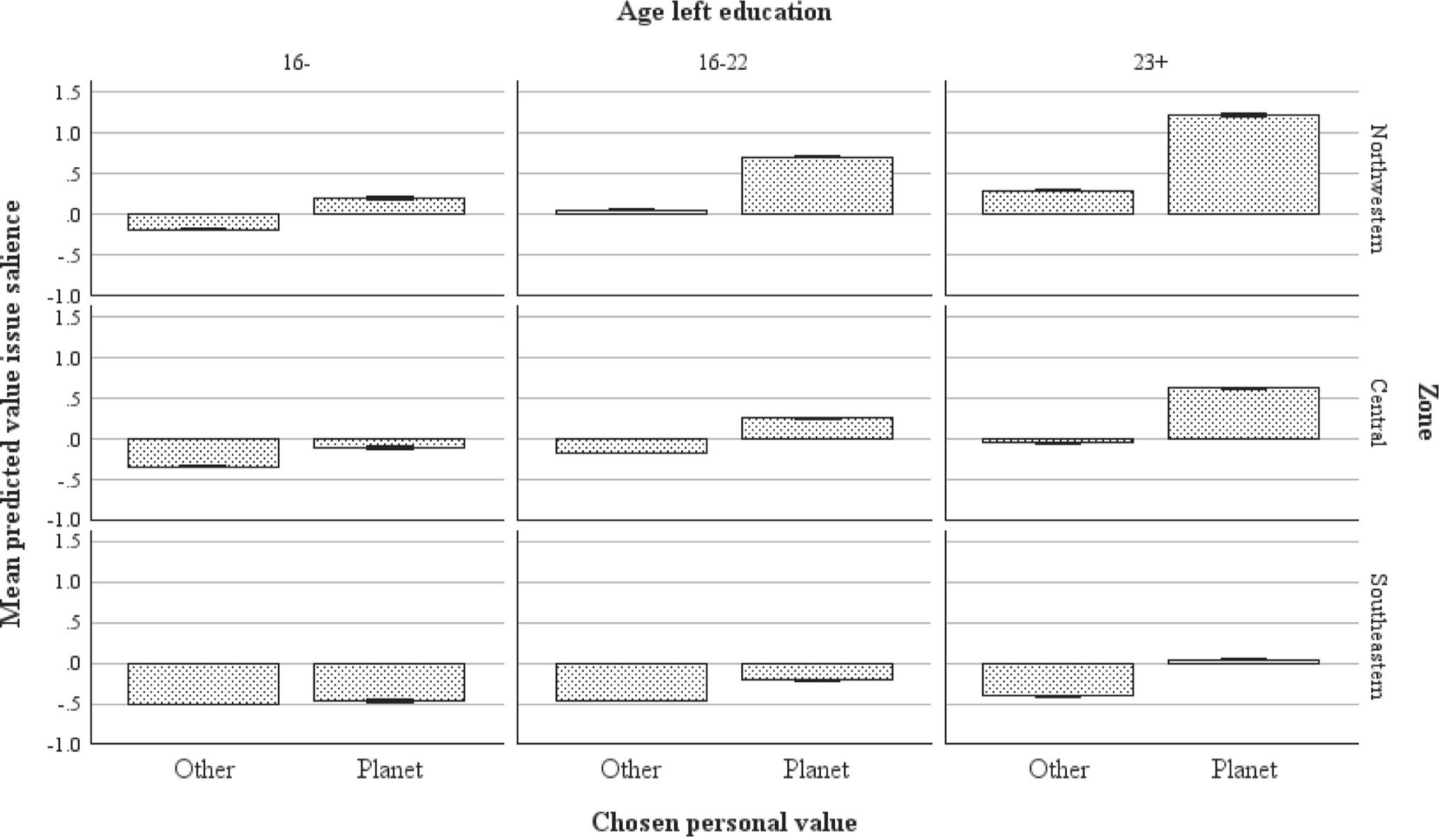
Association between Respect for the planet and green issue salience under different conditions of age left education and economic-cultural zone (bar charts with 95% CI)

**Fig. 2:**
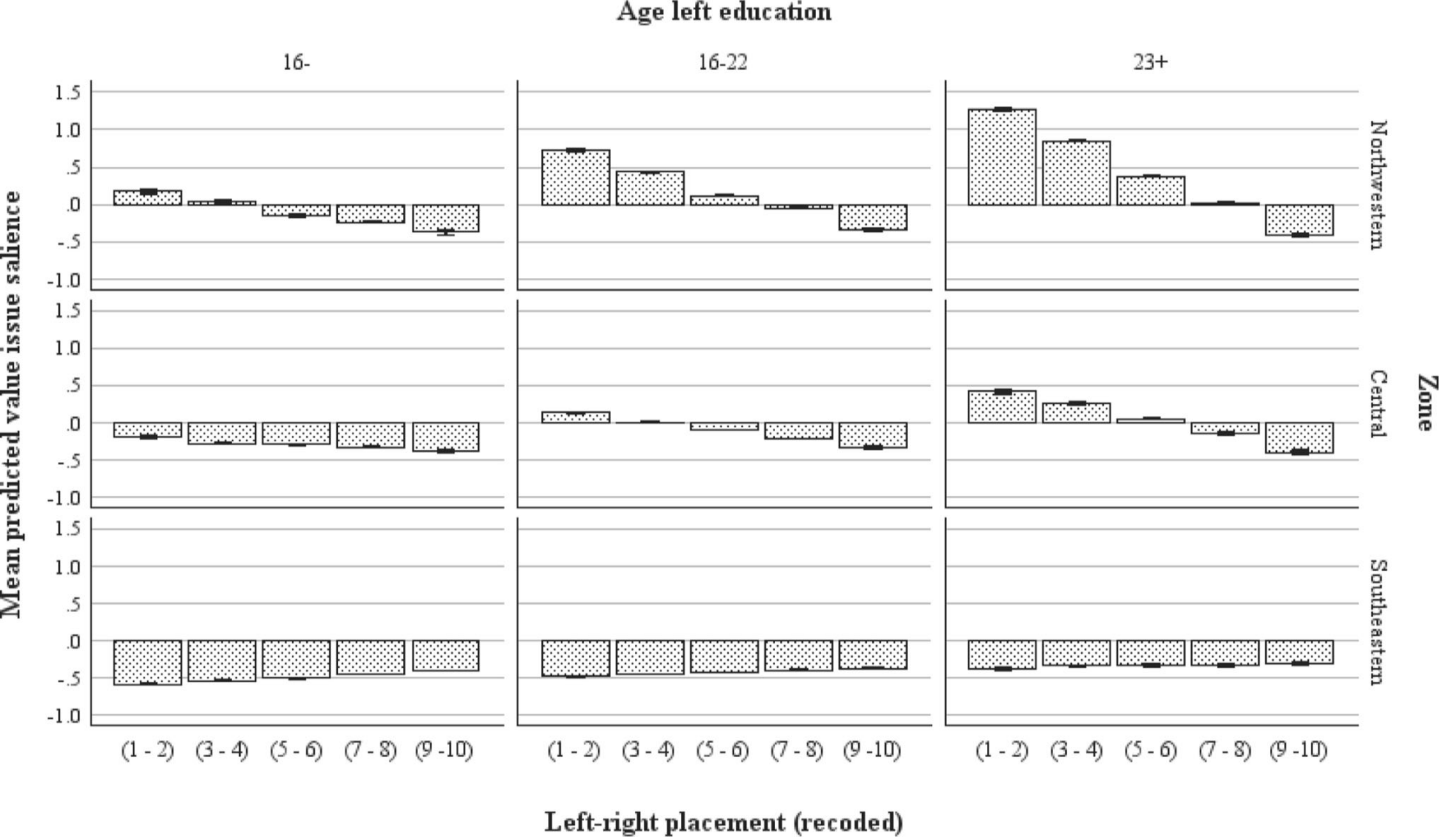
Association between left–right placement and green issue salience under different conditions of age left education and economic-cultural zone (bar charts with 95% CI)

The main results of the analysis are visualized in Figs. 1 and 2. Figure 1 gives an overview of the association between Respect for the planet and the predicted value of green issue salience under different conditions of age left education and zone, controlling for the other variables. A similar overview of the association between left–right placement and the predicted value of green issue salience is presented in Fig. 2. The details of the multilevel regression analysis are reported in Table 2. Due to the standardization of the variables, the mean value of green issue salience across the non-FC countries and survey years was 0 and the total variance 1. Model 1 in Table 2 shows the coefficients of the individual-level control variables in the regression if they are ≥|.10|. This applied to interview mode and the very small group (*N* = 157) of gender diverse persons. Also, there was a small decreasing tendency in green issue salience over the survey years. Model 2 shows that the participants who chose Respect for the planet had a substantial higher score on issue salience in standard deviation units (*B* = 0.58, 95% *CI* [0.57, 0.59], *p* < 0.001). Model 3 adds left–right placement, age left education, the interaction terms for age left education and Respect for the planet as well as for age left education and left–right placement, respectively. Model 3 shows that those who mentioned their political position had a somewhat higher green issue salience than the others, in particular, if they had left-leaning preferences, but not if they had right-leaning preferences. The country-level variables zone and EU membership were added in Model 4, indicating that green issue salience decreased from Northwest to Southeast, with a somewhat higher score in the EU member countries. Additionally, it was examined whether National affluence made a significant difference, but this was not the case (not shown). Model 5 reveals three significant interactions between zone and the variables Respect for the planet, left–right placement and age left education, respectively. Model 6 presents the three-way interaction between left–right placement, age left education and zone; the three-way interaction with Respect for the planet was not significant.

**Table 2:** Green issue salience as dependent variable in the multilevel regression analysis (non-FC countries)^a^). *p < 0.05, **p < 0.01, ***p < 0.001. ^a^All variables, except the dichotomous variables and survey year, have been standardized (mean score in non-FC countries = 0, *SD* = 1). ^b^The coefficients of the individual-level control variables (gender categories, age categories, living in a rural or urban environment, interview mode) were only reported if their absolute value is ≥ .10

	Regression coefficients and 95% CI					
	Model 1 (control)	Model 2 (add Respect for the planet)	Model 3 (add left–right placement)	Model 4 (add country-level variables)	Model 5 (interactions with zone)	Model 6 (3-way interaction)
Intercept	0.04 [− 0.11, 0.19]	− 0.04 [− 0.18, 0.09]	− 0.14 [− 0.26, − 0.02]	− 0.27 [− 0.45, − 0.09]	− 0.29 [− 0.47, − 0.12]	− 0.29 [− 0.47, − 0.12]
*Individual level*						
Respect for the planet		0.58 [0.57, 0.59]***	0.48 [0.47, 0.50]***	0.48 [0.47, 0.50]***	0.45 [0.44, 0.47]***	0.45 [0.44, 0.47]***
Age left education			0.08 [0.07, 0.08]***	0.08 [0.07, 0.08]***	0.08 [0.07, 0.09]***	0.08 [0.07, 0.09]***
Dummy for political placement			0.12 [0.11, 0.14]***	0.12 [0.11, 0.14]***	0.12 [0.10, 0.13]***	0.12 [0.10, 0.13]***
Left–right placement			− 0.15 [− 0.16, − 0.15]***	− 0.15 [− 0.16, − 0.15]***	− 0.15 [− 0.16, − 0.15]***	− 0.15 [− 0.15, − 0.14]***
*Country level*						
Zone				− 0.18 [− 0.26, − 0.10]***	− 0.18 [− 0.25, − 0.10]***	− 0.17 [− 0.25, − 0.10]***
EU Member				0.18 [− 0.02, 0.38]	0.21 [0.01, 0.40]*	0.21 [0.01, 0.40]*
*Interactions*						
Planet*education			0.17 [0.15, 0.18]***	0.17 [0.15, 0.18]***	0.14 [0.12, 0.15]***	0.13 [0.12, 0.15]***
Left–right*education			− 0.08 [− 0.09, − 0.08]***	− 0.08 [− 0.09, − 0.08]***	− 0.05 [− 0.06, − 0.05]***	− 0.05 [− 0.06, − 0.05]***
Planet*zone					− 0.10 [− 0.12, − 0.08]***	− 0.10 [− 0.11, − 0.08]***
Left–right*zone					0.09 [0.09, 0.10]***	0.09 [0.09, 0.10]***
Education*zone					− 0.02 [− 0.02, − 0.01]***	− 0.02 [− 0.03, − 0.01]***
Left–right*education* zone						0.02 [0.02, 0.03]***
*Control variables*^b^*)*						
Dummy gender diverse	0.25 [0.09, 0.40]**	0.20 [0.05, 0.36]*	0.08 [− 0.08, 0.23]	0.07 [− 0.08, 0.23]	0.05 [− 0.10, 0.20]	0.05 [− 0.10, 0.20]
Interview mode (online)	0.18 [0.16, 0.20]***	0.15 [0.13, 0.17]***	0.14 [0.12, 0.16]***	0.14 [0.12, 0.16]***	0.15 [0.13, 0.17]***	0.15 [0.13, 0.17]***
Survey year	− 0.02 [− 0.03, − 0.02]***	− 0.02 [− 0.03, − 0.02]***	− 0.03 [− 0.03, − 0.02]***	− 0.03 [− 0.03, − 0.02]***	− 0.02 [− 0.03, − 0.02]***	− 0.02 [− 0.03, − 0.02]***
*Coefficients of Determination*						
Pseudo R2 (marginal)	0.01	0.06	0.11	0.17	0.18	0.18
Pseudo R2 (conditional)	0.12	0.15	0.18	0.20	0.21	0.21

Zooming in on the upper right-hand corner of both figures (i.e., those who lived in the non-FC countries of the Northwest and left their education at 23 or more) enables a more detailed analysis of the relationships between the variables. This involves eight countries and 21,709 participants. Of these 25% choose Respect for the planet. The predicted value of green issue salience in this group was almost 1 *SD* higher than that of the others (see Fig. 1). The left-leaning participants had about the same score, but in this case the right-leaning participants made a difference in negative direction (see Fig. 2). Put differently, the correlation between Respect for the planet and green issue salience was *r* = 0.32, between left–right placement and green issue salience *r* = − 0.36, and between Respect for the planet and left–right placement *r* = − 0.19 (all *p*’s < 0.001). Inspection of the bar charts (not shown) further supported the observation that the relationships between the two variables and green issue salience were relatively independent of each other. In other words, although many right-leaning participants tended to choose another value than Respect for the planet, those who did so had higher scores on green issue salience than those who did not.

### Moderating impact of education in the FC countries

Although the reliability of the summed the green issue salience items in the FC countries was marginal, a multilevel regression analysis was possible (without the variable left–right placement). The analysis in Table 3 is largely equivalent with Model 2 of Table 2. In this case also, Respect for the planet had a higher score on issue salience. Again, there were positive coefficients for age left education and the interaction between Respect for the planet and age left education. The participants in the EU member states had a slightly higher green issue salience score than the others. The extremely small group (*N* = 56) of gender diverse persons and those who were interviewed online had a higher score. Again, there was a small decreasing tendency in green issue salience over the survey years. Hence, despite its limitations, Table 3 largely resembled the results of Table 2.

**Table 3:** Green issue salience as dependent variable in the multilevel regression analysis (FC countries)^a^). *p < 0.05, **p < 0.01, ***p < 0.001. ^a^See Table 2. ^b^See Table 2

Regression coefficients and 95% CI	Model
Intercept	0.00 [− 0.09, 0.08]
*Individual level*	
Respect for the planet	0.37 [0.35, 0.40]***
Age left education	0.04 [0.03, 0.05]***
*Country level*	
EU Member	0.12 [0.01, 0.22]*
*Interaction*	
Planet * education	0.13 [0.11, 0.16]***
*Control variables*^b^)	
Dummy gender diverse	0.56 [0.28, 0.84]***
Interview mode (online)	0.21 [0.18, 0.24]***
Survey year	− 0.03 [− 0.03, − 0.03]***
*Coefficients of Determination*	
Pseudo R2 (marginal)	0.03
Pseudo R2 (conditional)	0.04

## Discussion

This study has significantly improved our understanding of Respect for the planet as a political value. The results indicate that Respect for the planet was significantly related to green issue salience and that the strength of this relationship varied under different conditions. A key condition was the interaction with age left education, used as a proxy for educational attainment. Another condition was the cultural, economic, and political context in which the participants lived. The combination of endorsing Respect for the planet, having a tertiary education and living in non-FC Northwestern countries was linked to a level of green issue salience that was about 1 *SD* higher than the mean in all non-FC countries. The mean green issue salience in FC countries was substantially lower than this, but even under this condition Respect for the planet and its interaction with education were linked to a level that was about 0.5 *SD* higher. In non-FC countries, the variable left–right political placement showed similar relationships and statistical interactions as Respect for the planet, but the two variables were relatively independent of each other.

In hindsight, the relevance of education and economic-cultural zone for green issue salience was, in fact, already suggested by the climate protests that started among students in Northwestern Europe (Baiardi and Morana 2021; Boulianne and Ohme 2022; Svensson and Wahlström 2023). Our results show systematically how education and economic-cultural zone were associated with the salience of green issues. It should be emphasized that education is not only a matter of knowledge and skills, but also of, what Meyer (2015) calls, socialization experiences to be concerned with overall social welfare and the environment. The interaction of education and economic-cultural zone suggests that the provision of these experiences is dependent on the cultural climate of a country. However, we do not have data that allow us to determine the specific underlying mechanism of this effect. The same applies to the moderating effects of education on the relationships between Respect for the planet and green issue salience, which is new, as well as the relationship between left–right political placement and green issue salience, which is in line with earlier work (Smith and Mayer 2019; Czarnek et al. 2021). Recent studies emphasize that tertiary education may provide people with knowledge and skills, such as systems thinking, that may create a “sustainability mindset” to guide their interpretations and actions in many settings (Cripps and Smith 2024). Such a mindset is deemed crucial for sustainable leadership development, but it should not be overlooked that having a secondary education can also make a positive difference for green issue salience.

The variable economic-cultural zone was associated with green issue salience and had statistical interactions with Respect for the planet and left–right placement. The interaction with left–right placement was found earlier (Smith and Mayer 2019; Czarnek et al. 2025), the interaction with Respect for the planet is new. Political polarization in affluent countries makes it important that Respect for the planet and left–right placement had relatively independent relationships with green issue salience. In the countries with the highest polarization (as indicated by the size of the correlation of left–right placement), a number of right-leaning participants chose Respect for the planet as a personal value, and they had higher scores on green issue salience than those who did not. This agrees with Lockwood (2018) who observed that right-wing opposition is directed less to the issue of climate change itself than to the perception of climate change as an issue on the agenda of a liberal, cosmopolitan elite.

The control variables did not play a large role. The survey year showed a slightly decreasing trend in green issue salience across the period 2019 to 2024. In hindsight, green issue salience was at its peak in about 2021. Interview mode (online) was associated with a somewhat larger green issue salience; online interviews were correlated with age left education (*r* = 0.20), which indicates that persons with tertiary education were over-represented in online panels The very small category of gender diverse persons had a relatively high level of green issue salience, both in non-FC and FC countries. The high score agrees with an earlier analysis (de Boer and Aiking 2025), suggesting that gender diverse persons may be more receptive to visions of a gender-equal and environmentally caring world than binary genders.

Overall, the conclusion may be drawn that Respect for the planet has the potential to become a core political value, although the prospects depend on the education level and the culture in a country. This would mean that the value can help in policy development and communication, in particular, to support the public legitimation of policy changes for environment and climate. A point in case is a recent policy experiment in Switzerland (one of the countries in Northwestern Europe), which provided the participants in the treatment group with the information that “*if every person in the world had the same impact on the environment as every person living in Switzerland, we would need around three planets*” (Presberger et al. 2023). In comparison with the control group, this brief factual information increased citizens’ knowledge and concern levels and resulted in small increases in support for import restrictions on environmentally harmful products. Although this result might seem a promising start, the small effects indicate that in addition to brief factual information, much more attention is necessary to the question of how policy changes can be legitimized. This question can be addressed systematically within a framework that van Leeuwen (2007) has synthesized, describing how legitimation is related to authority (including the authority of expertise), moral evaluation (based on values), rationality (based on theories of reality) and story logic (telling short, memorable stories, including myths or memes) (see e.g., de Boer and Aiking 2021; Olsson and Johansson 2025). Hence, references to the planet should be embedded in broader communications in order to contribute to the legitimation of changes.

### Limitations and further research

The study has several limitations, which mean that the results need to be interpreted with caution. A serious limitation is the dependence on a secondary analysis of data that was gathered for broader purposes than explaining green issue salience. However, strong points are the large number of observations and the robustness of the findings demonstrated by the similarities between non-FC and FC countries. Unfortunately, the data did not include situation-specific factors, such as the experience of climate extremes, which has been shown to increase the salience of climate change at a regional level (Hoffmann et al. 2022; Grechyna 2025). This factor may give a somewhat different view on the relationship between green issue salience and geographic contexts than the present study can offer. Also, the data did not include important control variables such as household income and perceived material costs of mitigation (Bush and Clayton 2023; Presberger et al. 2023). In countries where the polarization about climate change is high, the debate between climate skeptics and believers can be considered as an intergroup conflict, which requires group-related variables (Bliuc et al. 2015). Finally, Respect for the planet was measured in a single item and it is not clear how the participants interpreted this item, for instance, whether they considered the planet’s wellbeing as a living entity (Redvers 2021). Further research should improve this measure and consider its links with other variables such as care for nature (Schwartz and Cieciuch 2022), the personal attachment a person may have to nature (Richardson et al. 2022), and global place attachment (Devine-Wright et al. 2015). Future research should also address how references to the planet can be used in policy development and communication.

## Conclusions

The key message of this paper is that endorsement of the value Respect for the planet was associated with a higher level of green issue salience but that its potential value should not be considered in isolation. Its capacity came to the fore particularly in combination with an advanced education and a cultural context valuing autonomy. This is important because the abstract value needs to be interpreted in theory and practice, which is sensitive to processes that stimulate and empower citizens in developing thoughts and actions, such as through education and autonomy. Also important is that the association was relatively independent of the variable left–right placement. The association existed in a context of high political polarization and, to a certain extent, even under other less favorable conditions, such as living in a country with a Communist inheritance or having a preference for right-wing policies. These results strongly suggest that Respect for the planet has the potential to become a core political value that, in combination with education and appropriate cultural conditions, can support policymakers, educators, researchers, and the general public in the pursuit of environment and climate objectives, such as the legitimation of policy changes.

## Data Availability

The data are publicly available.

## References

[CR1] Akaliyski, P., C. Welzel, and J. Hien. 2022. A community of shared values? Dimensions and dynamics of cultural integration in the European Union. *Journal of European Integration* 44: 569–590. 10.1080/07036337.2021.1956915.

[CR2] Altman, I., and S. M. Low, eds. 1992. *Place attachment*. New York: Plenum Press.

[CR3] Baiardi, D., and C. Morana. 2021. Climate change awareness: Empirical evidence for the European Union. *Energy Economics* 96: 105163. 10.1016/j.eneco.2021.105163.

[CR4] Baranowski, M., R. A. Huber, P. Jabkowski, and J. Szulecka. 2025. Left–right political orientation fails to explain environmental attitudes of Europeans outside Western Europe: Exploring the moderating role of party positions and issue salience. *Environmental Politics* 34: 793–816. 10.1080/09644016.2024.2401257.

[CR5] Bevan, S., W. Jennings, and C. Wlezien. 2016. An analysis of the public’s personal, national and EU issue priorities. *Journal of European Public Policy* 23: 871–887. 10.1080/13501763.2015.1070191.

[CR6] Bliuc, A. M., C. McGarty, E. F. Thomas, G. Lala, M. Berndsen, and R. Misajon. 2015. Public division about climate change rooted in conflicting socio-political identities. *Nature Climate Change* 5: 226–229. 10.1038/nclimate2507.

[CR7] Boulianne, S., and J. Ohme. 2022. Pathways to environmental activism in four countries: Social media, environmental concern, and political efficacy. *Journal of Youth Studies* 25: 771–792. 10.1080/13676261.2021.2011845.

[CR8] Bush, S. S., and A. Clayton. 2023. Facing change: Gender and climate change attitudes worldwide. *American Political Science Review* 117: 591–608. 10.1017/S0003055422000752.

[CR9] Buzogány, A., and P. Scherhaufer. 2023. The new climate movement: Organization, strategy, and consequences. In *Routledge handbook of environmental policy*, ed. Jörgens, H., C. Knill, and Y. Steinebach, 358–380. London: Routledge.

[CR10] Clark, L. A., and D. Watson. 1995. Constructing validity: Basic issues in objective scale development. *Psychological Assessment* 7: 309–319. 10.1037/14805-012.

[CR11] Crawley, S., H. Coffé, and R. Chapman. 2022. Climate belief and issue salience: Comparing two dimensions of public opinion on climate change in the EU. *Social Indicators Research* 162: 307–325. 10.1007/s11205-021-02842-0.

[CR12] Cripps, K., and S. Smith. 2024. Embedding a sustainability mindset in responsible management education. *International Journal of Organizational Analysis* 32: 1522–1538. 10.1108/IJOA-05-2023-3774.

[CR13] Czarnek, G., M. J. Hornsey, S. Bucki, and M. Kossowska. 2025. As countries become more affluent, climate change attitudes are more politically polarised. *Journal of Environmental Psychology* 103: 102579. 10.1016/j.jenvp.2025.102579.

[CR14] Czarnek, G., M. Kossowska, and P. Szwed. 2021. Right-wing ideology reduces the effects of education on climate change beliefs in more developed countries. *Nature Climate Change* 11: 9–13. 10.1038/s41558-020-00930-6.

[CR15] de Boer, J., and H. Aiking. 2021. Favoring plant instead of animal protein sources: Legitimation by authority, morality, rationality and story logic. *Food Quality and Preference* 88: 104098. 10.1016/j.foodqual.2020.104098.

[CR16] de Boer, J., and H. Aiking. 2024. Citizens and conspiratorial anti-science beliefs: Opposition vs support in 38 countries across Europe. *Public Understanding of Science* 33: 1027–1045. 10.1177/09636625241245371. 38629712 10.1177/09636625241245371 PMC11505401

[CR17] de Boer, J., and H. Aiking. 2025. Measuring gender diversity in public surveys: Implications for environmental values and sustainable choices in Europe. *Ecological Economics* 238: 108743. 10.1016/j.ecolecon.2025.108743.

[CR18] Dennison, J. 2019. A review of public issue salience: Concepts, determinants and effects on voting. *Political Studies Review* 17: 436–446. 10.1177/1478929918819264.

[CR19] Devine-Wright, P., J. Price, and Z. Leviston. 2015. My country or my planet? Exploring the influence of multiple place attachments and ideological beliefs upon climate change attitudes and opinions. *Global Environmental Change* 30: 68–79. 10.1016/j.gloenvcha.2014.10.012.

[CR20] Donders, Y. 2020. Diversity in Europe: From pluralism to populism? In *European populism and human rights*, ed. J. Vidmar, 52–71. Brill: Leiden.

[CR21] Eckert, L., S. Stagl, and B. Schemel. 2025. Social acceptance of climate policies: Insights from Austria. *Ecological Economics* 237: 108708. 10.1016/j.ecolecon.2025.108708.

[CR22] European Commission. 2020. *Eurobarometer 92.4 (2019).* Brussels: Kantar Public [producer]. GESIS Data Archive, Cologne. ZA7602 Data file Version 1.0.0, 10.4232/1.13652.

[CR23] European Commission. 2021. *Eurobarometer 95.3, June-July 2021.* Brussels/Cologne: GESIS, ZA7783, dataset version 1.0.0, 10.4232/1.13826.

[CR24] European Commission. 2022. *Eurobarometer 97.5, June-July 2022.* Brussels/Cologne: GESIS, ZA7902 Data file Version 1.0.0, 10.4232/1.14010.

[CR25] European Commission. 2023. *Eurobarometer 98.2 (January-February 2023).* Brussels/Cologne: GESIS, ZA7953 Data file Version 1.0.0, 10.4232/1.14081.

[CR26] European Commission. 2024a. *Eurobarometer 99.4 (May-June 2023).* Brussels/Cologne: Kantar Public/GESIS, ZA7997 Data file Version 1.0.0, 10.4232/1.14167.

[CR27] European Commission. 2024b. *Eurobarometer 100.2 (October-November 2023).* Brussels/Cologne: GESIS, ZA8779 Data file Version 1.0.0, 10.4232/1.14363.

[CR28] European Commission. 2024c. *Eurobarometer 101.3 (April-May 2024).* Brussels/Köln: GESIS, ZA8843 Datenfile Version 1.0.0, 10.4232/1.14376.

[CR29] Fehlner, W. 2019. Educating for sustainability: The crucial role of the tertiary sector. *Journal of Sustainable Development* 12: 18–28. 10.5539/jsd.v12n2p18.

[CR30] Feitelson, E. 1991. Sharing the globe: The role of attachment to place. *Global Environmental Change* 1: 396–406. 10.1016/0959-3780(91)90005-E.

[CR31] Foret, F., and O. Calligaro. 2018. Analysing European values: An introduction. In *European values. Challenges and opportunities for EU governance*, ed. Foret, F., and O. Calligaro, 1–20. New York: Routledge.

[CR32] Fraembs, T. V., and S. Drobnič. 2024. Ill-informed or ideologically driven? Climate change awareness and denial in Europe. *Population and Environment* 46: 21. 10.1007/s11111-024-00462-7.

[CR33] Frischhut, M. 2022. Future direction of travel (De Lege Ferenda). In *The ethical spirit of EU values: Status Quo of the Union of values and future direction of travel*, ed. M. Frischhut, 219–240. Cham: Springer International Publishing.

[CR34] Grechyna, D. 2025. Raising awareness of climate change: Nature, activists, politicians? *Ecological Economics* 227: 108374. 10.1016/j.ecolecon.2024.108374.

[CR35] Haverland, M., M. De Ruiter, and S. Van de Walle. 2018. Agenda-setting by the European Commission. Seeking public opinion? *Journal of European Public Policy* 25: 327–345. 10.1080/13501763.2016.1249014.

[CR36] Hoffmann, R., R. Muttarak, J. Peisker, and P. Stanig. 2022. Climate change experiences raise environmental concerns and promote Green voting. *Nature Climate Change* 12: 148–155. 10.1038/s41558-021-01263-8.

[CR37] Hornsey, M. J. 2021. The role of worldviews in shaping how people appraise climate change. *Current Opinion in Behavioral Sciences* 42: 36–41. 10.1016/j.cobeha.2021.02.021.

[CR38] Huntington, S. P. 1993. The clash of civilizations? *Foreign Affairs* 72: 22–49.

[CR39] Inglehart, R. 2006. Mapping global values. *Comparative Sociology* 5: 115–136. 10.1163/156913306778667401.

[CR40] Inglehart, R., and W. E. Baker. 2000. Modernization, cultural change, and the persistence of traditional values. *American Sociological Review* 65: 19–51. 10.1177/000312240006500103.

[CR41] Jasanoff, S. 2010. A new climate for society. *Theory, Culture & Society* 27: 233–253. 10.1177/02632764093614.

[CR42] Kahan, D. M., E. Peters, M. Wittlin, P. Slovic, L. L. Ouellette, D. Braman, and G. Mandel. 2012. The polarizing impact of science literacy and numeracy on perceived climate change risks. *Nature Climate Change* 2: 732–735. 10.1038/nclimate1547.

[CR43] Knutsen, O. 1995. Value orientations, political conflicts and left-right identification: A comparative study. *European Journal of Political Research* 28: 63–93. 10.1111/j.1475-6765.1995.tb00487.x.

[CR44] Lee, H., C. Park, and H. Jung. 2024. The role of tertiary education on CO2 emissions: Evidence from 151 countries. *Environment, Development and Sustainability* 26: 32081–32103. 10.1007/s10668-024-04828-7.

[CR45] Lockwood, M. 2018. Right-wing populism and the climate change agenda: Exploring the linkages. *Environmental Politics* 27: 712–732. 10.1080/09644016.2018.1458411.

[CR46] Lorenzini, J., G.-A. Monsch, and J. Rosset. 2021. Challenging climate strikers’ youthfulness: The evolution of the generational gap in environmental attitudes since 1999. *Frontiers in Political Science* 3: 279. 10.3389/fpos.2021.633563.

[CR47] McCann, J. A. 1997. Electoral choices and core value change: The 1992 presidential campaign. *American Journal of Political Science* 41: 564–583. 10.2307/2111777.

[CR48] McCright, A. M., R. E. Dunlap, and S. T. Marquart-Pyatt. 2016. Political ideology and views about climate change in the European Union. *Environmental Politics* 25: 338–358. 10.1080/09644016.2015.1090371.

[CR49] Meyer, A. 2015. Does education increase pro-environmental behavior? Evidence from Europe. *Ecological Economics* 116: 108–121. 10.1016/j.ecolecon.2015.04.018.

[CR50] Milfont, T. L., E. Zubielevitch, P. Milojev, and C. G. Sibley. 2021. Ten-year panel data confirm generation gap but climate beliefs increase at similar rates across ages. *Nature Communications* 12: 4038. 10.1038/s41467-021-24245-y. 10.1038/s41467-021-24245-y PMC8260718 34230472

[CR51] Noy, S., and T. L. O’Brien. 2019. Science for good? The effects of education and national context on perceptions of science. *Public Understanding of Science* 28: 897–916. 10.1177/0963662519863575. 31354045 10.1177/0963662519863575

[CR52] Olsson, A., and J. Johansson. 2025. Legitimising different futures: Swedish forest management as a climate change mitigation measure. *Environmental Science & Policy* 171: 104174. 10.1016/j.envsci.2025.104174.

[CR53] Pickard, S. 2022. Young environmental activists and Do-It-Ourselves (DIO) politics: Collective engagement, generational agency, efficacy, belonging and hope. *Journal of Youth Studies* 25: 730–750. 10.1080/13676261.2022.2046258.

[CR54] Polak, R. 2023. Values: A contested concept. Problem outline and interdisciplinary approaches. In *Values–politics–religion: the European values study: In-depth analysis–interdisciplinary perspectives–future prospects*, ed. Polak, R., and P. Rohs, 33–93. Cham: Springer.

[CR55] Poortinga, W., C. Demski, and K. Steentjes. 2023. Generational differences in climate-related beliefs, risk perceptions and emotions in the UK. *Communications Earth & Environment* 4: 229. 10.1038/s43247-023-00870-x.

[CR56] Presberger, D., F. Quoß, L. Rudolph, and T. Bernauer. 2023. Factual information on the environmental impacts of consumption abroad increases citizens’ problem awareness, but not support for mitigating such impacts. *Environmental Science & Policy* 146: 101–112. 10.1016/j.envsci.2023.04.019.

[CR57] Redvers, N. 2021. The determinants of planetary health. *The Lancet Planetary Health* 5: e111–e112. 10.1016/S2542-5196(21)00008-5 33713610 10.1016/S2542-5196(21)00008-5

[CR58] Richardson, M., I. Hamlin, L. R. Elliott, and M. P. White. 2022. Country-level factors in a failing relationship with nature: Nature connectedness as a key metric for a sustainable future. *Ambio* 51: 2201–2213. 10.1007/s13280-022-01744-w. 35641693 10.1007/s13280-022-01744-w PMC9481760

[CR59] Rockström, J., J. F. Donges, I. Fetzer, M. A. Martin, L. Wang-Erlandsson, and K. Richardson. 2024. Planetary Boundaries guide humanity’s future on Earth. *Nature Reviews Earth & Environment* 5: 773–788. 10.1038/s43017-024-00597-z.

[CR60] Sagiv, L., and S. H. Schwartz. 2022. Personal values across cultures. *Annual Review of Psychology* 73: 517–546. 10.1146/annurev-psych-020821-125100. 10.1146/annurev-psych-020821-125100 34665670

[CR61] Schwartz, S. H. 2014. National culture as value orientations: Consequences of value differences and cultural distance. In *Handbook of the Economics of Art and Culture* 547–586. Amsterdam: Elsevier.

[CR62] Schwartz, S. H. 2015. Cultural values influence and constrain economic and social change. In *Culture matters—in Russia and everywhere: Backdrop for the Russia-Ukraine conflict*, ed. Y. Y. L. E. Harrison, 287–302. London: Lexington Books.

[CR63] Schwartz, S. H., G. V. Caprara, and M. Vecchione. 2010. Basic personal values, core political values, and voting: A longitudinal analysis. *Political Psychology* 31: 421–452. 10.1111/j.1467-9221.2010.00764.x.

[CR64] Schwartz, S. H., and J. Cieciuch. 2022. Measuring the refined theory of individual values in 49 cultural groups: Psychometrics of the revised portrait value questionnaire. *Assessment* 29: 1005–1019. 10.1177/1073191121998760. 33682477 10.1177/1073191121998760 PMC9131418

[CR65] Simister, J. 2014. Delayed effects of education on graduate earnings: A degree of hope. *Global Journal of Human and Social Sciences* 14: 33–41.

[CR66] Smith, E. K., and A. Mayer. 2019. Anomalous Anglophones? Contours of free market ideology, political polarization, and climate change attitudes in English-speaking countries, Western European and post-Communist states. *Climatic Change* 152: 17–34. 10.1007/s10584-018-2332-x.

[CR67] Steenkamp, J. B. E. 2019. Global versus local consumer culture: Theory, measurement, and future research directions. *Journal of International Marketing* 27: 1–19. 10.1177/1069031X18811289.

[CR68] Svensson, A., and M. Wahlström. 2023. Climate change or what? Prognostic framing by Fridays for Future protesters. *Social Movement Studies* 22: 1–22. 10.1080/14742837.2021.1988913.

[CR69] Taber, K. S. 2018. The use of Cronbach’s alpha when developing and reporting research instruments in science education. *Research in Science Education* 48: 1273–1296. 10.1007/s11165-016-9602-2.

[CR70] Tam, K. P., and T. L. Milfont. 2020. Towards cross-cultural environmental psychology: A state-of-the-art review and recommendations. *Journal of Environmental Psychology* 71: 101474. 10.1016/j.jenvp.2020.101474.

[CR71] van Leeuwen, T. 2007. Legitimation in discourse and communication. *Discourse & Communication* 1: 91–112. 10.1177/1750481307071986.

[CR72] Waytz, A., R. Iyer, L. Young, J. Haidt, and J. Graham. 2019. Ideological differences in the expanse of the moral circle. *Nature Communications* 10: 4389. 10.1038/s41467-019-12227-0. 10.1038/s41467-019-12227-0 PMC6763434 31558713

[CR73] Weber, W., and W. E. Saris. 2015. The relationship between issues and an individual’s left–right orientation. *Acta Politica* 50: 193–213. 10.1057/ap.2014.5.

[CR74] Welsch, H. 2022. What shapes cognitions of climate change in Europe? Ideology, morality, and the role of educational attainment. *Journal of Environmental Studies and Sciences* 12: 386–395. 10.1007/s13412-021-00745-7. 35036279 10.1007/s13412-021-00745-7 PMC8753329

[CR75] Yearley, S. 2024. Non-governmental organizations and the environmental movement: Challenges in climate change framing. In *Climate, Science and Society: A primer*, ed. Baker, Z., T. Law, M. Vardy, and S. Zehr, 77–85. London: Routledge.

[CR76] Žalėnienė, I., and P. Pereira. 2021. Higher education for sustainability: A global perspective. *Geography and Sustainability* 2: 99–106. 10.1016/j.geosus.2021.05.001.

